# Mindful Sensation Seeking: An Examination of the Protective Influence of Selected Personality Traits on Risk Sport-Specific Stress

**DOI:** 10.3389/fpsyg.2019.01719

**Published:** 2019-08-08

**Authors:** Marie Ottilie Frenkel, Joana Brokelmann, Arne Nieuwenhuys, Robin-Bastian Heck, Christian Kasperk, Martin Stoffel, Henning Plessner

**Affiliations:** ^1^Institute of Sports and Sports Sciences, Heidelberg University, Heidelberg, Germany; ^2^Psychological Institute, Goethe University Frankfurt, Frankfurt, Germany; ^3^Department of Exercise Sciences, The University of Auckland, Auckland, New Zealand; ^4^Behavioural Science Institute, Radboud University, Nijmegen, Netherlands; ^5^Department of Internal Medicine I and Clinical Chemistry, Steroid Laboratory, University Hospital Heidelberg, Heidelberg, Germany; ^6^Institute of Medical Psychology, University Hospital Heidelberg, Heidelberg, Germany

**Keywords:** mindfulness, anxiety, cortisol, sensation seeking, risk sport-specific stress

## Abstract

Stress often has a negative influence on sports performance. Stress-induced decreases in performance can be especially disastrous for risk sports athletes, who often put their life at risk when practicing their sport. Therefore, it is of great importance to identify protective factors in stressful situations in risk sports. On average, risk sports athletes score extremely high on the personality trait sensation seeking. At the same time, theoretical considerations about dispositional mindfulness suggest that mindful athletes can handle stress more effectively. The main goal of this experiment is to examine the influence of sensation seeking and mindfulness on the stress response to a risk sport-specific stressor. To induce stress, 88 male students completed the Heidelberg Risk Sport-Specific Stress Test (HRSST) which utilizes fear of falling as the stressful event during a climbing exercise. Psychological (anxiety) and physiological (cortisol) responses were measured at multiple time points before and after the HRSST to determine the severity of the stress response. In reaction to the stressor, a significant increase in self-reported state anxiety, but no significant increase in cortisol were observed. The mindfulness subscale *external observation* correlated positively with anxiety in the climbing wall, sensation seeking and the anxiety scales after the jump correlated negatively and sensation seeking predicted anxiety subscales after the jump in hierarchical regression analyses. However, mindfulness did not predict anxiety measures. Neither sensation seeking nor mindfulness correlated significantly with cortisol levels. The results suggest that high sensation seekers perceive a risk sport-specific stressor as less stressful. The missing physiological response might be explained by the Cross-Stressor-Adaptation-Hypothesis and particularities of the sample. Good internal observers might be especially aware of their need of stimulation and new experiences, which in turn might explain the higher experience-seeking scores. Future studies should further examine the role of mindfulness in stressful situations and the interaction of its subscales with sensation seeking. The current experiment offers new possibilities for adjoining research fields at the interface between sports sciences, psychology and medicine: The findings can be transferred to high risk professions such as police officers, firefighters and military forces (e.g., for selection processes or for interventions).

## Introduction

Athletes plunge from mountains only wearing a wingsuit, free climbers scale high rock faces without any form of protection, and surfers aim to ride huge waves before they break the shores. High risk sports athletes who practice those demanding activities frequently set their physical integrity at risk, making it crucial to deliver peak-performance. Typically, highly demanding situations induce distress and therefore threaten peak performance-delivery (Paulus et al., 2009; Röthlin et al., 2016). In contrast, however, some risk sports athletes are known to report positive rather than negative responses and emotions during task execution (Arijs et al., 2017; Frenkel et al., 2018b; Houge Mackenzie and Brymer, 2018), often resulting in peak performance. Why do some people report not being afraid in such extreme situations? Empirical studies point to specific personality traits that may influence stress and performance in (high risk) sports (Plessner et al., 2009; Allen et al., 2013; Röthlin et al., 2016). Pertinent to the current study, one of these traits – sensation seeking – is prevalent among risk sports athletes and seems to have stress-buffering and performance-facilitating effects (Anshel and Anderson, 2002; Ruedl et al., 2012; Frenkel et al., 2018b). In addition to being high in sensation seeking, narrative research indicates that risk sports athletes describe their strengths in risky situations in words that resemble mindful mindsets (Arijs et al., 2017; Houge Mackenzie and Brymer, 2018). Based on these data, an intriguing question is whether dispositional mindfulness may contribute to risk sports athletes’ positive emotional responses and functioning in highly demanding situations. Building on existing narrative research, the aim of the present study is to provide an experimental examination of the protective influence of sensation seeking and mindfulness on risk sport-specific stress responses.

### Stress

According to Lazarus and Folkman’s (1984) transactional model of stress, stress results from the athlete’s subjectively perceived discrepancy between the demands being placed by the environment and coping resources available in a particular situation. High risk sports athletes are often required to respond to situations which threaten their physical integrity or psychological well-being (Breivik, 1999b). In such circumstances, they usually have little opportunity to make corrective decisions, for example, deciding to interrupt a first free-solo ascent in rock climbing to try again later, or correcting errors and avoiding structures while flying in a wingsuit at a speed of over 200 mph. When an individual perceives environmental demands to outweigh their coping resources, a negative and unpleasant psychological state ensues, characterized by feelings of stress and anxiety (Lazarus, 2000). In this respect, anxiety is regarded as an aversive emotional and motivational state that arises when facing uncertainty or a perceived existential threat (Eysenck et al., 2007).

Critical incidents in high risk sports hold high levels of novelty, uncontrollability and personal threat of injury or death (Breivik, 1999b). Besides showing a psychological response (as indicated above), the human body also shows a physiological response to such situations (Campbell and Ehlert, 2012), including activation of the fast reacting sympathetic adrenomedullary system (SAM) – which triggers the release of adrenaline and noradrenaline – and the slower hypothalamic-pituitary-adrenal (HPA) axis, which triggers the release of cortisol from the adrenal cortex. Indeed, several studies have shown that critical incidents place high physiological demands on athletes. For example, although physiological response patterns were slightly inconsistent across studies, it has been found that athletes showed increases in subjective stress (e.g., self-reported anxiety) and salivary cortisol in response to various (sport-specific) experimental stress protocols (Lautenbach et al., 2014; Lautenbach, 2017; Frenkel et al., 2018b).

In general, both psychological and physiological stress responses are associated with impairments in cognitive performance (Eysenck et al., 2007) as well as with a decrease in sports performance (Lautenbach et al., 2014; Frenkel et al., 2018b; e.g., see Nieuwenhuys and Oudejans, 2012, 2017, for a review).

### Sensation Seeking

With regard to high risk sports, one personality trait that may protect risk sports athletes from negative effects of stress is “sensation seeking” (Zuckerman, 1994, 1996). Indeed, risk sports athletes have been shown to score extremely high on measures of sensation seeking (Breivik, 1999b; Ruedl et al., 2012) which is defined as the “seeking of varied, novel, complex, and intense sensations and experiences and the willingness to take physical, social, legal, and financial risks for the sake of such experience” (Zuckerman, 1994, p. 27).

According to the psychobiological model of sensation seeking (Zuckerman, 1994, 1996), individuals differ in their optimal levels of physiological arousal and the stimulation required to establish a certain level of arousal. In contrast to low sensation seekers (LSS) – who feel better in less stimulating environments –, high sensation seekers (HSS) tend to have lower baseline levels of dopamine and norepinephrine, which leads these individuals to continuously seek new and intense sensations to maintain their optimal levels of arousal (Zuckerman, 2007).

In the context of the transactional model of stress (Lazarus and Folkman, 1984), HSS may be hypothesized to differ from LSS with respect to (a) their primary appraisal of the performance environment (e.g., lower perceived demands); (b) their perceived ability to cope (e.g., more resources) and, hence, may be expected to (c) show reduced psychological responses (e.g., lower levels of anxiety), as well as (d) reduced physiological responses (e.g., lower levels of salivary cortisol).

Indeed, with regards to (a), Franken et al. (1992) showed that HSS in comparison to LSS tended to judge risky and dangerous situations as less threatening and therefore postulate negative outcomes as less likely to occur. With regard to (b), empirical studies in sports are more scarce. Nevertheless, one study with high school athletes confirmed sensation seeking as a stress-resiliency factor, with HSS reporting better stress management coping skills than LSS (Smith et al., 1992). Regarding psychological responses (c), little is known about the relationship between sensation seeking and anxiety. Examining a sample of university students, Franken et al. (1992) found sensation seeking to be negatively correlated with anxiety. However, in a sample of parachute jumpers, only one out of four subscales of sensation seeking correlated negatively with state anxiety (Breivik et al., 1998) and – more recently – in a sample of 30 sports students, HSS did not show significantly lower anxiety than LSS in response to a high risk sport-specific stressor (Frenkel et al., 2018b). Finally, regarding physiological responses (d), high sensation seeking has been found to be related to lower baseline levels of cortisol (Shabani et al., 2011) and an attenuated cortisol response to stress (Couture et al., 2008), indicating that HSS might tolerate new, potentially stressful experiences better than LSS. Breivik (1999a) did not find a significant correlation between cortisol and sensation seeking in a sample of extreme sports athletes. However, in a recent experimental study, Frenkel et al. (2018b) confirmed that HSS showed lower levels of cortisol in response to a risk sport-specific stressor than LSS. Taken together, these data (“a,” “b,” “c,” and “d”) indicate that HSS appraise demanding performance environments more positively and exhibit psychological and physiological responses that allow them to perform better in stressful, high risk sports situations than LSS.

### Mindfulness

In contrast to being reckless, risk sports athletes describe the use of internal strategies in risky situations that appear to reflect mindful mindsets (Arijs et al., 2017; Houge Mackenzie and Brymer, 2018): Including high present-moment awareness, high attunement with the environment, a simultaneous internal focus, as well as the use of deliberate value-guided action. Through well-tuned knowledge of their own physical and psychological capacities and limitations, risk sports athletes’ actions are often guided by a “leave your ego at the door” mentality (Arijs et al., 2017, p. 7). Hence, in absence of (high levels of) anxiety – which often serves as a natural “brake” on behavior in LSS – HSS’ mindful mindsets may allow them to make required corrective decisions when conditions are unsafe or suddenly turn aversive (such as the wind in the wrong direction for BASE jumpers).

Mindfulness is considered a specific kind of attention direction (Kabat-Zinn, 1994). Following an operational definition given by Bishop et al. (2004), mindfulness can be divided in two components: The process of continuous direction of attention and the inner attitude with which this process is carried out (openness, acceptance, self-support). A central feature of mindfulness is “centering” in the presence. Mindfulness directly impacts human behavior by interrupting automatized reaction patterns and by replacing them with flexible actions appropriate for the respective situation (Kabat-Zinn, 1994).

Different competing approaches attempt to explain positive mechanisms of trait mindfulness on well-being and health (Shapiro et al., 2006; Brown et al., 2007; Creswell and Lindsay, 2014; Creswell et al., 2019). Based on this work, and in context of the transactional model of stress (Lazarus and Folkman, 1984), highly mindful in contrast to lowly mindful individuals may be hypothesized to (a) show more favorable appraisals of their performance environment, (b) evaluate their coping resources more positively, and – hence – exhibit (c) reduced psychological responses (e.g., lower levels of anxiety), and (d) reduced physiological responses (e.g., lower levels of salivary cortisol).

Indeed, with regards to (a), the mindfulness stress buffering account (Creswell and Lindsay, 2014; Creswell et al., 2019) states that trait mindfulness mitigates stress assessment because stressors are observed with acceptance and equanimity which, in turn, buffers primary threat appraisals. In line with this assumption, Brown et al. (2012a) demonstrated that mindfulness may buffer attentional reactivity to threatening stimuli. Regarding (b), through the buffering of primary threat appraisals, mindfulness should facilitate positive secondary appraisals in favor of coping resources, decrease subsequent rumination, and increase effective coping strategies (Creswell and Lindsay, 2014). Mindful persons are found to possess better emotion-regulation abilities: Negative emotions are avoided less often (Shapiro et al., 2006). Moreover, as negative states are avoided only to a minor extent, a stronger voluntary exposition to negative emotions like anxiety takes place. As a result, a desensitization concerning anxiety responses occurs (Brown et al., 2007; Shapiro et al., 2006). In the context of sports, Josefsson et al. (2017) showed indirect effects of dispositional mindfulness on coping via rumination and emotion regulation. In line with these effects, regarding (c), research confirms that mindfulness is associated with reduced (i.e., less negative) psychological responses to stress. Outside the context of sports, research using different approaches (correlational, quasi-experimental, and laboratory studies) has shown that trait mindfulness is related to decreased levels of trait and state anxiety (e.g., Brown and Ryan, 2003; Arch and Craske, 2010). Within the sports context, Röthlin et al. (2016) showed that in elite athletes, trait mindfulness is negatively related to cognitive competitive trait anxiety, thereby helping them to perform better. Finally, regarding the physiological response to stress (d), the mindfulness stress buffering account (Creswell and Lindsay, 2014; Creswell et al., 2019) suggests that mindful individuals should exhibit increased activation of regulatory pathways in the prefrontal cortex, whilst reducing bottom-up stress-reactivity (e.g., HPA axis responses), thus inhibiting cortisol production and release from the adrenal cortex. Recent laboratory studies with healthy participants seem to confirm these assumptions (Arch and Craske, 2010; Brown et al., 2012b; Manigault et al., 2018). However, in the context of sports, there is a lack of experimental studies investigating the link between trait mindfulness and cortisol in response to a sport-specific stressor.

### The Current Study

Integrating the literature, sensation seeking and trait mindfulness are described as personality characteristics that could potentially contribute to effective stress regulation and performance in demanding (high risk) sports situations (Kabat-Zinn et al., 1985; Smith et al., 1992; Breivik, 1999a; Röthlin et al., 2016; Frenkel et al., 2018b). However, there is a shortage of experimental studies in the context of sports and, to our knowledge, there is no research investigating the role of sensation seeking AND mindfulness within one study. Against this background – and based on Lazarus and Folkman’s (1984) transactional model of stress –, the present experimental study investigates how sensation seeking and mindfulness affect individuals’ psychological (anxiety) and physiological (cortisol) response to a sport-specific stressor and whether mindfulness can explain additional variance in the stress response beyond sensation seeking.

• Hypothesis (H) 1a and b: Sensation seeking is negatively associated with (a.) state anxiety and (b.) salivary cortisol, in response to the Heidelberg Risk Sport-Specific Stress Test (HRSST).
• H2a and b: Mindfulness is negatively associated with (a.) state anxiety and (b.) salivary cortisol, in response to the HRSST.
• H3a and b: Sensation seeking explains a significant proportion of variance in (a.) state anxiety and (b.) salivary cortisol, in response to the HRSST.
• H4a and b: Beyond sensation seeking, mindfulness explains a significant proportion of variance in the prediction of (a.) state anxiety and (b.) salivary cortisol, in response to the HRSST.

## Materials and Methods

### Participants

During pre-selection, a sample of *N* = 207 male sports students of the Heidelberg University (*M* = 22.9, *SD* = 3.3) were screened for eligibility based on exclusion criteria that included sports habits and health condition. Participants were excluded from the study when they had more than 5 h of climbing experience (*n* = 68; to maximize the effectiveness of the stress induction; Ogden, 2012), or reported a particular fear of heights (*n* = 17), consumed medication containing cortisol (*n* = 1), or had injuries (*n* = 14). If none of the exclusion criteria were met, participants were invited for the experiment. Nineteen persons were not available or refused to participate.

Following the screening process, 88 male students, aged between 18 and 31 (*M* = 22.5, *SD* = 2.8), were deemed eligible and agreed to participate in this study. Two persons had to be excluded from the entire analyses because one participant did not jump and the other consumed branched amino acids and creatine during the experiment.

The most frequently reported types of sports in the sample (multiple answers allowed) were soccer (21.9%), fitness (12.5%), and weight training (10.2%). Concerning high risk sports, 13 persons had engaged in downhill mountain biking, three persons had done a bungee jump, three persons had engaged in different kinds of surfing activities and one person had done skydiving before. For medium risk sports, martial arts and American Football/rugby were mentioned by one participant, respectively, and skiing was reported by two participants. On average, the participants had 8.6 h of sports practice per week (*SD* = 3.7) and rated their fitness on average as 71.0 on a scale ranging from 1 to 100 (*SD* = 18.3). Participants gave written informed consent and were compensated for their participation (15 euros). The procedures were approved by the local Ethics Commission, a university board associated with the Faculty of Behavioural and Cultural Studies.

### Design

**FIGURE 1:**
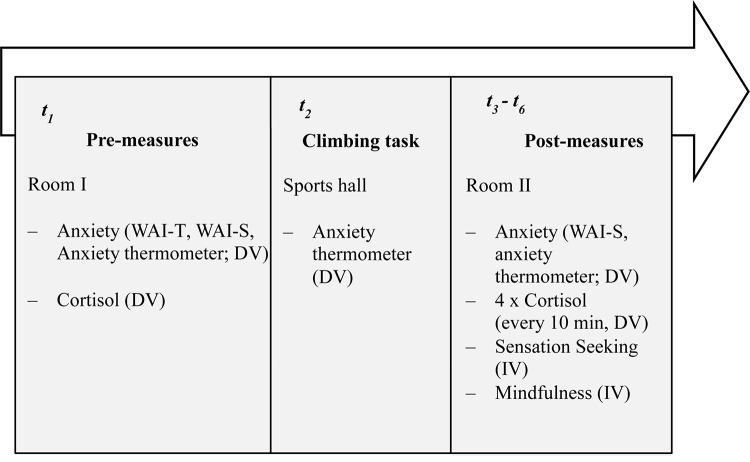
Graphical summary of the procedure, measures, and measurement points. IV: independent variable, DV: dependent variable.

This study is embedded in a bigger between-within-subject-design-study (Experiment 3 of Frenkel, 2018). In this study, participants were randomly assigned to an ego depletion vs. control group, while their stress parameters were assessed multiple times in the course of the investigation (see Figure 1). A state of ego depletion was induced using a 10-min copying task (Bertrams et al., 2010). Participants in the ego depletion group were instructed to copy a text about the history of the German city of Mannheim as fast and error-free as possible, while leaving out the letters “e” and “n”. Because these two letters appear frequently in the German language, this variation of the copying task can be considered strenuous. Participants in the control group had to copy the text without leaving out any letters. They were also instructed to copy the text as fast and error-free as possible. Effects of the ego depletion are presented elsewhere (Frenkel, 2018). The analyses were conducted with both the experimental and the control group and the authors controlled for the influence of the experimental condition (see below).

### Procedure

In this study, the psychological and physiological responses to a sport-specific stressor were tested at six measurement points (for an overview see Figure 1). To adequately represent the situational demands in risk sports, we conducted the HRSST (Frenkel et al., in press), which has been introduced as an innovative, externally valid and standardized stress induction protocol. This protocol uses a climbing task with a subsequent “jump into the rope” that leads to a fall of about 3 m to induce stress. Participants were asked not to consume caffeine, juices, food, nicotine or alcoholic beverages within 1 h prior to the experiment. In accordance with the recommendations of Kudielka et al. (2009), the effect of circadian hormone rhythms was minimized by holding testing time relatively constant and conducting all sessions in the afternoon (between 2 and 6 p.m.). Based on the idea that the stress induction and, consequently, the increase in cortisol are maximal for unpredictable tasks (Kirschbaum et al., 1993), baseline measurements before the HRSST were taken in a room outside the sports hall. This set-up ensured that participants did not develop any expectations about the upcoming climbing task. For reasons of standardization, the study followed a written protocol that described both the test procedure as well as the instructions given by the investigators.

At the first point of measurement (*t*1), participants filled out questionnaires for state and trait anxiety, the first salivary sample (baseline measurement) was taken and participants completed the copying task to manipulate their self-control strength. Then, participants were led to the sports hall where they put on a harness and received instructions for the HRSST: They were instructed to climb to the top of the wall top-rope secured (12 m). Having reached the top of the wall, participants reported their current anxiety state. Then the belayer explained that they had to “jump into the rope”,^1^ resulting in a fall of about 2–3 m. The instruction was to jump backward, not to touch the rope, and to land with both feet simultaneously on the wall. The participants were instructed to choose the moment of the jump themselves. The jump (or fall) was extended by the belayer by loosening the rope in the moment of the fall. If participants displayed at least three of five previously defined abort criterions at this point (i.e., shaking of the legs, slowdown and solidification of movements, cramping, loud and panting breathing and repeated asking), the study was stopped. After the jump, participants were lowered by the belayer.

Finally, the participants were led to the third room where the remaining measurements were taken. At the moment of the participant’s jump into the rope, the investigator started a digital stopwatch (CASIO, HS-3V-1RET) so that, after the jump, four salivary samples could be taken with 10-min intervals (*t*3–*t*6). Moreover, at each time point, participants again filled out questionnaires regarding state anxiety (WAI-S; Ehrlenspiel et al., 2009). After the final measurement (i.e., at *t*6), participants completed questionnaires to assess both personality traits (e.g., Mindfulness: Kentucky Inventory for Mindfulness Skills Short; Höfling et al., 2011; Sensation Seeking: SSS-V; Zuckerman, 1994; German version: Beauducel et al., 2003). After the study, participants were thanked, compensated, and fully debriefed.

### Measures

#### Predictors

##### Sensation seeking

Sensation seeking was assessed with the Sensation Seeking Scale V (SSS-V; Zuckerman, 1994, German version: Beauducel et al., 2003). The instrument consists of 40 items in a forced-choice format (e.g., “I would like to try to surf”. vs. “I would not like to try to surf”.) of which ten items can be allocated to one of the following for facets, respectively: 1. *Thrill and Adventure Seeking* (*TAS*): Search for danger and adventures, 2. *Experience Seeking* (*ES*): Search for experiences through a non-conformist lifestyle, 3. *Disinhibition* (*DIS*): Tendency for disinhibition in social situations, 4. *Boredom Susceptibility* (*BS*): Aversion to repetition and routines. Besides the separate scores for each facet, a total score varying between min = 0 and max = 40 can be calculated. Based on the current sample, the questionnaire showed satisfactory to good internal consistency, with values similar to those reported for the norm sample (i.e., total scale: α = 0.82; subscales between 0.64 and 0.81; cf. Beauducel et al., 2003).

##### Mindfulness

Mindfulness was measured using the Kentucky Inventory for Mindfulness Skills Short (KIMS-D Short; German version: Höfling et al., 2011). The 20 items of the short version are to be answered on a five-point Likert-type scale (ranging from 1 = *never or seldom* to 5 = *very often or always*) with participants rating to which extent the statements applied to them. The items can be allocated to the subscales *observation of external phenomena* (*obs-ext*), *observation of internal phenomena* (*obs-int*), *describing* (*des*), *acting mindfully* (*AM*) and *accepting without rating* (*AWR*; Höfling et al., 2011). An example item for the *AM* scale is “I judge whether my thoughts are good or bad”. The five scales were combined to an index for mindfulness (in the following called mindfulness index). For mindfulness measured with the KIMS-D Short (Höfling et al., 2011), all reliabilities were satisfactory to good (i.e., α = 0.74 for *observing* to α = 0.85 for *describing*) and comparable to norm-values reported in the literature (i.e., α = 0.70–0.89; Höfling et al., 2011).

#### Psychological Stress Response

##### State anxiety

Participants’ psychological response to the stressor was assessed using two measures of state anxiety: Firstly, it was assessed at two measurement points (*t*1, *t*3), using the German questionnaire Wettkampf-Angst-Inventar-State (WAI-S; Ehrlenspiel et al., 2009). The WAI-S consists of 12 items (four-point scale, from 0 = “*not at all*” to 3 = “*extremely*”) and the subscales *somatic anxiety* (*som*), *cognitive anxiety* (*cog*) and *confidence* (*conf*). An example item for the *somatic anxiety* subscale is “In the present moment… my heart throbs”. The internal consistencies of the WAI-S were found to be clearly (at *t*1, before the stress induction) and slightly (at *t*3, after the stress induction) lower in the present sample (between α*_*cog*_* = 0.52 and α*_*conf*_* = 0.79), compared to the norm sample (Ehrlenspiel et al., 2009: between α*_*cog*_* = 0.79 and α*_*conf*_* = 0.82). Secondly, state anxiety was assessed at three measurement points (*t*1, *t*2, *t*3) using an anxiety thermometer (Houtman and Bakker, 1989). The anxiety thermometer captures the current feelings of anxiety by one item asking the question “How do you rate your current feelings of anxiety?” on a 10-cm visual analog scale, ranging from 0 = *no anxiety at all* to 10 = *extreme anxiety*. Houtman and Bakker (1989) report test-retest reliabilities of 0.60–0.70.

In this experiment, we applied two different measures of state anxiety at the same time. The questionnaire WAI-S permits with its three subscales a more detailed view of the facets of anxiety while at the same time taking more time to fill it out. The one-item anxiety thermometer promised a weaker reliability, but a better handling during the climbing task (see Nieuwenhuys et al., 2008).

#### Physiological Stress Response

##### Cortisol

The physiological stress response was assessed repeatedly using the cortisol concentration in saliva. Participants’ samples were collected using Salivette Blue^®^ Device (Sarstedt GmbH, Nümbrecht). Thereby, participants chewed on a synthetic swab for 1 min. As the cortisol peaks about 20 min after experiencing the stressor (the jump) (Kirschbaum and Hellhammer, 2000; Campbell and Ehlert, 2012), saliva samples were taken five times in 10-min intervals after the stress induction (10, 20, 30, and 40 min after the jump). The measurement point of interest was *t*4, 20 min after the jump. Additionally, the return to baseline levels could be assessed from the later measurement points. The saliva samples were stored at –20°C until analyses. The biochemical analysis of the samples was conducted by the steroid laboratory of the Steroid Laboratory, University Hospital Heidelberg. After thawing, the samples were centrifuged at 3000 rpm for 5 min which resulted in a clear supernatant of low viscosity. Salivary concentrations were determined using chemiluminescence immunoassay with high sensitivity (IBL International, Hamburg, Germany). The intra- and inter-assay coefficients for cortisol, which express the precision or repeatability of immunoassay test results, were good (i.e., below 8%; cf. Schultheiss and Stanton, 2009).

#### Control Variables

##### Trait anxiety

The control variable trait anxiety was measured with the German questionnaire Wettkampf-Angst-Inventar-Trait (WAI-T; Brand et al., 2009). The inventory consists of 12 items (four-point scale, from 0 = “*not at all*” to 3 = “*extremely*”). The 12 items can be allocated to the scales *somatic anxiety* (*som*), *cognitive anxiety* (*cog*) and *concentration difficulties* (*conc*). The items of the WAI-T are similar to those of the WAI-S, however, the introductory formulation “In the present moment…” is replaced by “Before sporting challenges…”. In line with Smith et al.’s (1990) Sport Anxiety Scale – on which the WAI-T was based – and to optimize statistical power of our regression models, WAI-T subscales were combined to arrive at a single trait anxiety score for each individual (in the following called “WAI-T index”).^2^ In general, internal consistency of the WAI-T was acceptable to good (with αs between 0.60 and 0.82) and comparable to original values reported by Brand et al. (2009); i.e., with αs between 0.77 and 0.81).

### Data Processing and Statistical Analyses

Initially, the data of all variables were analyzed to detect any missing and extreme values. Because missing values only occurred occasionally (≤5%) and unsystematically, they were replaced using the *expectation maximization* (*EM*) method (Wirtz, 2004; Tabachnick and Fidell, 2007). Concerning cortisol, missing values were replaced using multiple imputations. Beforehand, necessary conditions were checked separately for each group using *missing-completely-at-random* (*MCAR*) tests (Tabachnick and Fidell, 2007). To identify extreme values, boxplots were created separately for experimental groups (depletion and non-depletion) and measurement points. *Tukey-far-out* was chosen as a criterion for extreme values: Values which are more than the triple interquartile range above/under the 75%/25% quartile were identified as extreme values (Tabachnick and Fidell, 2007). Concerning cortisol, the exclusion criterion was a distance of ±3 SDs above/under the average group value (Adam and Kumari, 2009).

After the data had been prepared, it was checked for normal distribution as a necessary condition for the following arithmetical analyses. As the Kolmogorov–Smirnov test easily detects a violation of the normal distribution in big samples, additionally, histograms were used. If the Kolmogorov–Smirnov test was found to be significant, analyses were conducted nonetheless because of its high sensitivity and non-parametric relations were calculated on a descriptive statistical level additionally.

To identify the covariates, the dependent variables (i.e., state anxiety and cortisol) were correlated with possible covariates, including age, fitness, previous climbing experience, trait anxiety and experimental condition (ego depletion vs. no ego depletion). In addition, as a possible covariate for cortisol, starting time (time of day) was coded as a variable to control for the influence of the decrease in cortisol during the day. Beforehand, the covariate was adjusted for extreme values following the procedure described above.

Cortisol values were checked substantially and arithmetically for plausibility. To display the change in the response to the stressor, different characteristic values were calculated. Firstly, the increase from baseline to the jump was calculated by taking the difference between *t*1 and *t*4 (i.e., 20 min after the jump), after values had been approximated to the normal distribution using the box-cox-transformation (Miller and Plessow, 2013). In addition, two different versions of the *area under the curve* (AUC) were calculated, the *area under the curve with respect to ground* (AUCg) and the *area under the curve with respect to increase* (AUCi; Pruessner et al., 2003). The AUCg is considered as an indicator for the absolute cortisol concentration over time, mapping the total area of trapezes between the measurement points. For the AUCi, the area between the first measurement point (baseline) and the zero point is subtracted from AUCg. AUC_i_ thus represents the change in cortisol over time compared to the baseline. If the value is positive, an increase occurs. If the AUCi value is negative, cortisol decreases.

To test hypotheses 1 and 2, (partial) correlations between the independent and the dependent variables (while controlling for covariates) were calculated. The correlations are based on *z*-standardized variables.

To test hypotheses 3 and 4, hierarchical regressions were calculated, using SS and mindfulness (plus the covariates) as predictors and anxiety (WAI-S at *t*1 and *t*3, anxiety thermometer at *t*1, *t*2, and *t*3) and cortisol (AUCg, AUCi, increase and cortisol at *t*4) as criteria. Doing so, covariates were entered in step 1, followed by sensation seeking in step 2 and mindfulness in step 3. To increase the interpretability of the residues, predictors were grand-mean centered (Field, 2009). The covariates age and previous climbing experience (in hours) were not centered because a useful unit already existed.

The assessment of statistical significance followed conventional criteria. A probability of *p* < 0.10 was considered as marginally significant, of *p* < 0.05 as significant, of *p* < 0.01 as highly significant and of *p* < 0.001 as extremely significant. IBM SPSS Statistics 24 was used for all statistical analyses.

## Results

### Data Preparation

Initially, the cortisol values were checked for plausibility: All values that were located outside the area that can be assessed for analyses using the assay (0.414–41.4 nmol/l) were excluded. This exclusion affected three participants for *t*1 and two participants for *t*3. Missing values were replaced using multiple imputation: This affected three participants for *t*1 and four participants for *t*3. In total, seven participants had to be excluded from the study because of content-related reasons or an extreme value at the third measurement point.

As there were no significant correlations between the experimental condition (ego depletion vs. no ego depletion) and the criteria (anxiety/cortisol), this potential covariate was not included into the regression analyses. Based on the significant correlations (see Supplementary Material 1), the covariates age, fitness state and climbing frequency as well as the WAI-T index were included in the respective analyses.

### Time Course Analyses

To indicate the overall effectiveness of the stress manipulation (i.e., regardless of sensation seeking and mindfulness), differences in state anxiety and cortisol before and after the climb (see Table 1) were compared across all participants using paired *t*-tests. In response to the stressor, on a psychological level, anxiety (as indexed by the anxiety thermometer) increased significantly over time (baseline *t*1 compared to *t*2 on top of the climbing wall) by an average of 2.73 points [i.e., 10-point scale; *SD* = 2.32; *Min* = −1, *Max* = 9; *t*(80) = −10.73, *p* < 0.001; *d* = 0.95]. On a physiological level, salivary cortisol concentrations (*t*1 compared to *t*4) showed a slight but non-significant increase by an average of 0.79 nmol/l [*SD* = 4.85; *Min* = −16.60, *Max* = 12.22; *t*(80) = −1.51, *p* = 0.13; *d* = 0.17].

**TABLE 1:** Time course analysis.

**Variables**	***M* (*SD*) at *t*1**	***M* (*SD*) at *t*2**	***M* (*SD*) at *t*3**	***M* (*SD*) at *t*4**
WAI-S som	1.48 (0.44)	–	1.91 (0.49)	–
WAI-S cog	1.34 (0.36)	–	1.26 (0.33)	–
WAI-S conf	2.94 (0.48)	–	3.03 (0.62)	–
Anxiety thermometer	0.8 (0.8)	3.5 (2.4)	1.3 (1.6)	–
Salivary cortisol (in nmol/l)	9.42 (9.96)	–	–	10.21 (7.40)

*WAI-S, Wettkampf-Angst-Inventar-State; WAI-S som, WAI-S somatic anxiety; WAI-S cog, WAI-S cognitive anxiety; WAI-S conf, WAI-S confidence.*

### Correlations (Hypotheses 1 and 2)

As appears from Table 2, measures of sensation seeking (SS scale) and mindfulness (KIMS) were not significantly correlated, *r*(82) = 0.11, *p* = 0.33.

**TABLE 2:** (Partial) correlations of the independent variables.

**Variables**	**1**	**2**	**3**	**4**	**5**	**6**	**7**	**8**	**9**	**10**	**11**
1. SS total	–	0.67^∗∗∗^	0.73^∗∗∗^	0.74^∗∗∗^	0.63^∗∗∗^	0.11	0.23^*^	0.10	0.00	0.03	–0.05
2. SS BS	–	–	0.32^∗∗^	0.38^∗∗∗^	0.17	0.11	0.22^*^	0.09	0.03	0.01	–0.07
3. SS Dis	–	–	–	0.34^∗∗^	0.22^*^	–0.03	0.13	–0.01	–0.11	–0.01	–0.06
4. SS ES	–	–	–	–	0.42^∗∗∗^	0.17	0.20	0.14	0.04	0.00	0.06
5. SS TAS	–	–	–	–	–	0.08	0.08	0.08	0.06	0.09	–0.04
6. KIMS index	–	–	–	–	–	–	0.66^∗∗∗^	0.53^∗∗∗^	0.45^∗∗∗^	0.47^∗∗∗^	0.48^∗∗∗^
7. KIMS obs-ext	–	–	–	–	–	–	–	0.52^∗∗∗^	–0.09	–0.06	0.03
8. KIMS obs-int	–	–	–	–	–	–	–	–	–0.09	0.25^*^	0.08
9. KIMS des	–	–	–	–	–	–	–	–	–	0.09	0.31^∗∗^
10. KIMS am	–	–	–	–	–	–	–	–	–	–	–0.05
11. KIMS awr	–	–	–	–	–	–	–	–	–	–	–

SS = Sensation Seeking; SS BS = SS Boredom-Susceptibility; SS Dis = SS Disinhibition; SS ES = SS Experience Seeking; SS TAS = SS Thrill-And-Adventure-Seeking; KIMS = Kentucky Inventory of Mindfulness Skills; KIMS obs-ext = KIMS observation-of-external-phenomena; KIMS obs-int = KIMS observation-of-internal-phenomena; KIMS des = KIMS describing; KIMS am = KIMS acting-mindfully; KIMS awr = KIMS accepting-without-rating. ^*^p < 0.05. ^∗∗^p < 0.01. ^∗∗∗^p < 0.001.

Regarding Hypothesis 1a, sensation seeking (SS total score) did not significantly correlate with any of the anxiety measures at baseline (i.e., *t*1; see Table 3). After the climb, however, sensation seeking was negatively correlated with the WAI-S somatic scale at *t*3 [*r*(82) = −0.22, *p* = 0.01] and with the WAI-S cognitive scale at *t*3 [*r*(82) = −31, *p* = 0.01]. No significant correlations of the SS total score were found with the WAI-S somatic scale at *t*3 as well as with the anxiety thermometer at *t*2 or *t*3. Regarding Hypothesis 1b, no relationship was observed between sensation seeking and cortisol responses (see Table 3).

**TABLE 3:** (Partial) correlations of the independent variables with the psychological and physiological dependent variables.

**Variables**	**WAI-S som *t*1**	**WAI-S cog *t*1**	**WAI-S conf *t*1**	**Anxiety thermometer *t*1**	**Anxiety thermometer *t*2**	**WAI-S som *t*3**	**WAI-S cog *t*3**	**WAI-S conf *t*3**	**Anxiety thermometer *t*3**	**AUC_g_**	**AUC_i_**	**Rise**	**Cortisol at *t*4**
SS total	0.12	0.04	–0.06	–0.16	–0.17	−0.22^*^	–0.31^∗∗^	0.15	0.00	–0.04	–0.02	–0.03	–0.04
BS	0.22^*^	–0.01	–0.06	–0.06	–0.15	–0.18	–0.11	–0.03	–0.11	0.06	–0.07	–0.07	–0.04
Dis	0.02	–0.03	–0.09	–0.13	–0.03	–0.02	–0.07	0.01	0.03	0.08	0.04	0.04	0.01
ES	0.02	–0.02	–0.11	–0.19	−0.22^*^	–0.10	−0.24^*^	0.19^*^	–0.08	–0.07	0.03	–0.004	–0.03
TAS	–0.02	0.19	–0.11	–0.19	−0.27^*^	−0.29^*^	–0.21	0.12	−0.26^*^	0.004	–0.07	–0.05	–0.09
KIMS index	–0.01	–0.11	−0.22^*^	–0.05	0.01	–0.04	–0.09	–0.16	0.01	–0.07	0.08	0.07	0.01
obs-ext	–0.12	–0.12	–0.05	–0.08	−0.25^*^	–0.20	–0.01	–0.14	−0.23^*^	0.09	–0.12	–0.12	–0.10
obs-int	0.12	–0.08	–0.03	–0.03	–0.08	–0.07	–0.05	–0.06	−0.27^*^	–0.01	–0.04	0.02	0.02
des	–0.08	–0.05	–0.32^∗∗^	0.002	0.12	0.03	–0.07	−0.25^*^	0.24^*^	–0.07	0.20	0.13	0.08
am	–0.01	–0.09	–0.21	–0.05	–0.02	0.06	–0.09	–0.16	0.11	–0.11	–0.16	–0.09	–0.11
awr	0.02	0.08	–0.17	0.09	0.09	0.06	0.04	–0.13	0.13	–0.04	0.16	0.07	0.10

SS = Sensation Seeking; SS DS = SS Boredom-Susceptibility; SS Dis = SS Disinhibition; SS ES = SS Experience Seeking; SS TAS = SS Thrill-And-Adventure-Seeking; KIMS = Kentucky Inventory of Mindfulness Skills; KIMS obs-ext = KIMS observation-of-external-phenomena; KIMS obs-int = KIMS-observation-of-intemal-phenomena; KIMS des = KIMS describing; KIMS am = KIMS acting-mindfully; KIMS awr = KIM accepting-without-rating; WAI-S = Wettkampf-Angst-Inventar-State; WAI-S som = WAI-S somatic-anxiety; WAI-S cog = WAI-S cognitive anxiety; WAI-S conf = WAI-S confidence; AUC_*g*_ = Area under the curve with respect to ground; AUC_*i*_ = Area under the curve with respect to increase. ^*^p < 0.05. ^∗∗^p < 0.01. ^∗∗∗^p < 0.001.

Regarding Hypothesis 2a, mindfulness (KIMS index) was negatively correlated with one component of anxiety (i.e., WAI-S confidence scale) at baseline [i.e., *t*1; *r*(92) = −22, *p* = 0.02]. No other significant correlations were found. Regarding Hypothesis 2b, no relationship was observed between mindfulness and cortisol responses (see Table 3).

In addition to the above, various correlations were found between psychological and physiological responses (see Table 4): AUCi correlated significantly positively with the anxiety thermometer at *t*1 [*r*(79) = 0.27, *p* = 0.02] and the anxiety thermometer at *t*2 [*r*(79) = 0.27, *p* = 0.02]. The cortisol value at *t*4 [*r*(79) = 0.27, *p* = 0.02] and the increase in cortisol [*r*(79) = 0.23, *p* = 0.04] each displayed a significant positive correlation with the anxiety thermometer at *t*2. No significant associations were found between the remaining dependent variables (see Table 4). At a physiological data level, no significant relationship was found between AUCi and AUCg (*r* = 0.10, *p* = 0.40). The reason may be that, as argued by Pruessner et al. (2003), the variables mirror differential aspects of the physiological response. Moreover, AUCg was not correlated significantly with the increase in cortisol [*r*(79) = 0.11, *p* = 0.33] but displayed a significant positive correlation with the cortisol value at *t*4 [*r*(79) = 0.84, *p* < 0.001].

**TABLE 4:** (Partial) correlations of the psychological and physiological dependent variables.

**Variables**	**1**	**2**	**3**	**4**	**5**	**6**	**7**	**8**	**9**	**10**	**11**	**12**	**13**
1. WAI-S som *t*1	–	0.26^*^	0.08	0.28^∗∗^	–0.01	0.21	0.23^*^	0.03	0.05	0.00	0.04	0.03	0.05
2. WAI-S cog *t*1	–	–	−0.22^*^	0.15	0.04	0.01	0.34^∗∗^	0.49^∗∗∗^	0.09	0.13	0.01	0.00	0.06
3. WAI-S conf *t*1	–	–	–	−0.28^*^	0.12	0.04	–0.17	0.49^∗∗∗^	0.29^∗∗^	0.16	–0.06	–0.06	0.03
4. Anxiety thermometer t_1_	–	–	–	–	0.24^*^	0.21	0.26^*^	−0.21^*^	0.43^*^	0.04	0.27^*^	0.22	0.19
5. Anxiety thermometer t_2_	–	–	–	–	–	0.49^∗∗∗^	0.27^∗∗^	0.01	0.61^∗∗^	0.19	0.27^*^	0.23^*^	0.27^*^
6. WAI-S som *t*3	–	–	–	–	–	–	0.39^∗∗∗^	–0.10	0.56^∗∗^	–0.04	0.15	0.13	0.10
7. WAI-S cog *t*3	–	–	–	–	–	–	–	–0.38^∗∗∗^	0.54^∗∗^	–0.12	0.21	0.16	0.13
8. WAI-S conf *t*3	–	–	–	–	–	–	–	–	0.32^∗∗^	0.11	–0.06	–0.08	–0.00
9. Anxiety thermometer t_3_	–	–	–	–	–	–	–	–	–			0.35^∗∗^	0.18
10. AUC_g_	–	–	–	–	–	–	–	–	–	–	0.10	0.10	0.84^∗∗∗^
11. AUC_i_	–	–	–	–	–	–	–	–	–	–	–	0.88^∗∗∗^	0.86^∗∗∗^
12. rise *t*1–*t*4	–	–	–	–	–	–	–	–	–	–	–	–	0.89^∗∗∗^
13. Cortisol *t*4	–	–	–	–	–	–	–	–	–	–	–	–	–

WAI-S = Wettkampf-Angst-Inventar-State; WAI-S som = WAI-S somatic-anxiety; WAI-S cog = WAI-S cognitive anxiety; WAI-S conf = WAI-S confidence; AUC_*g*_ = area under the curve with respect to ground; AUC = area under the curve with respect to increase. ^*^p < 0.05. ^∗∗^p < 0.01. ^∗∗∗^p < 0.001.

### Hierarchical Regression Analyses (Hypotheses 3 and 4)

Regarding Hypothesis 3a, after controlling for covariates, sensation seeking (SS total score) did not predict any of the WAI-S anxiety measures at baseline (i.e., *t*1), but was found to be a marginally significant predictor of anxiety as measured with the anxiety thermometer (see Table 5). After the climb (i.e., at *t*3), sensation seeking (SS total score) again marginally predicted anxiety as measured with the anxiety thermometer and could explain 26.8% of the total variance in WAI-S somatic [*R*^2^ = 0.27, Δ*R*^2^ = 0.07, *F*(3,84) = 11.60, *p* < 0.001] and 21.6% of the variance in WAI-S cognitive [*R*^2^ = 0.22, Δ*R*^2^ = 0.08, *F*(3,84) = 24.50, *p* < 0.001]. For the confidence component, SS was found to be a marginally significant predictor (see Table 5). Regarding Hypothesis 3b, no significant associations were observed between sensation seeking (SS total score) and any of the physiological variables (i.e., AUCg, AUCi, increase in cortisol and cortisol at *t*4).

**TABLE 5:** Hierarchical regressions of the dependent variables on SS and KIMS and the respective covariates.

**Dependent variable**	**Covariate(s), independent variables**	**Standardized beta**	***𝚫R*^2^**	***P***	***f*^2^**
WAIS som *t*1	WAIT index	0.46^∗∗∗^	0.22	0.00	0.28
	SS-total	0.02	0.00	0.85	0.00
	KIMS-index	0.01	0.00	0.94	0.00
WAIS cog *t*1	WAIT-index	0.49^∗∗∗^	0.22	0.00	0.28
	SS-total	0.04	0.00	0.68	0.00
	KIMS-index	–0.06	0.01	0.47	0.01
WAlS conf *t*1	WAIT-index	0.26^∗∗∗^	0.18	0.00	0.22
	SS-total	0.04	0.00	0.87	0.00
	KIMS-index	0.13^*^	0.04	0.04	0.04
Anxiety	WAIT-index	0.39^∗∗∗^	0.13	0.00	0.15
thermometer *t*1	SS-total	–0.18	0.03	0.08	0.03
	KIMS-index	–0.00	0.00	0.98	0.00
Anxiety	Climbing	−0.25^*^	0.09	0.02	0.10
thermometer *t*2	SS-total	–0.17	0.03	0.11	0.03
	KIMS-index	–0.09	0.01	0.41	0.01
WAIS som *t*3	WAIT-index	0.44^∗∗∗^	0.17	0.00	0.20
	Climbing	–0.17	0.05	0.09	0.05
	SS-total	–0.27^∗∗^	0.07	0.01	0.08
	KIMS-index	–0.02	0.00	0.84	0.00
WAIS cog *t*3	WAIT-index	0.45^∗∗∗^	0.15	0.00	0.18
	SS-total	–0.29^∗∗^	0.08	0.00	0.09
	KIMS-index	–0.02	0.00	0.86	0.00
WAIS conf *t*3	WAIT-index	0.32^∗∗^	0.12	0.00	0.14
	SS-total	–0.19	0.03	0.07	0.03
	KIMS-index	0.18	0.03	0.09	0.03
Anxiety	WAIT-index	0.40^∗∗∗^	0.13	0.00	0.15
thermometer *t*3	Climbing	–0.18	0.06	0.08	0.06
	SS-total	–0.20	0.04	0.05	0.04
	KIMS-index	–0.09	0.01	0.37	0.01

SS = Sensation Seeking; WAI-S = Wettkampf-Angst-Inventar-State; WAI-S som = WAI-S somatic-anxiety; WAI-S cog = WAI-S cognitive anxiety; WAI-S conf = WAI-S confidence; KIMS = Kentucky Inventory of Mindfulness Skills. ^*^p < 0.05. *^∗∗^p <* 0.01. ^∗∗∗^*p* < 0.001. *A posteriori* power analyses: small (*f*^2^ = 0.02), medium (*f*^2^ = 0.15), large effects (*f*^2^ = 0.35); α err prob < 0.05.

Regarding Hypothesis 4a, after controlling for covariates and sensation seeking, mindfulness (KIMS index) significantly predicted WAI-S confidence at baseline [β = 0.13, *t*(83) = 2.11, *p* = 0.04]. Together, the three predictors could explain 22.3% of the total variance [*R*^2^ = 0.22, Δ*R*^2^ = 0.04, *F*(3,84) = 8.03, *p* < 0.001]. After the climb (i.e., at *t*3), mindfulness was found to be a marginally significant predictor of WAI-S confidence, but not for the two other components of the WAI-S (Table 5). Mindfulness did not predict anxiety as measured with the anxiety thermometers at any measurement point (see Table 5). Regarding Hypothesis 4b, no significant associations were observed between mindfulness (KIMS index) and any of the physiological variables (i.e., AUCg, AUCi, increase in cortisol and cortisol at *t*4).

## Discussion

The current study investigated whether sensation seeking and mindfulness affect individuals’ psychological (anxiety) and physiological (cortisol) response to a sport-specific stressor: The HRSST. It was hypothesized that both sensation seeking and mindfulness would negatively correlate with anxiety (H1a and H2a) and cortisol (H1b and H2b), that sensation seeking would be a significant predictor of anxiety and cortisol in response to the HRSST (H3a and H3b), and that – beyond sensation seeking – mindfulness would explain additional variance in anxiety and cortisol (H4a and H4b).

### Psychological Response to the HRSST

#### Sensation Seeking

Our findings partly confirm hypotheses H1a and H3a that sensation seeking (SS) would be associated with and significantly predict participants’ psychological response to the HRSST. At *t*3, we found a significant negative relationship between SS and two of the subscales of state anxiety (the somatic and the cognitive component). In the corresponding regression analyses (i.e., predicting both anxiety components at *t*3) – and after controlling for covariates – SS explained a significant proportion of variance, with individuals scoring high on SS exhibiting lower levels of anxiety. As became apparent from the analyses of subscales, the observed effects are likely driven by the TAS component and the ES component of SS (see Table 3). This observation is in line with a study conducted by Breivik (1999a), in which the difference in SS between high risk sports athletes and sport science students was also caused by TAS and ES. As such, TAS and ES appear to be essential components of SS and its effect on individuals’ psychological response to risk-full sport situations. The current results are in line with the psychobiological multilevel theory (Zuckerman, 1994) and show that HSS differ from LSS in their psychological response to an unexpected stimulus, in this case the jump into the rope.

Contradicting H1a and H3a, SS was neither significantly associated with nor significantly predicted the confidence component of state anxiety. Whilst speculative, this may relate to the nature of the current task (i.e., a wall climb followed by a so-called jump in the rope), with which (a) participants had very little or no experience; and which (b), allowed participants little control over the course of action. Potentially, with higher levels of task experience or in tasks that allow more control, sensation seeking may also boost self-confidence and further contribute to the positive appraisal of high-risk performance environments that is characteristic of HSS. Future research aiming to investigate this matter may find Jones’ (1995) control model of anxiety – which distinguishes between intensity and direction (i.e., positive vs. negative) of the anxiety response – to be a useful framework.

Matching the effects observed with the WAI-S (somatic and cognitive anxiety subscale), SS was found to explain a significant proportion of variance in participants’ scores on the anxiety thermometer at *t*3. Unexpectedly, the effect of SS on anxiety thermometer scores at *t*2 failed to reach significance (*p* = 0.11, see Table 5). With the effect of SS on anxiety being generally small (see Table 5), one explanation for the absence of this effect may be that – being a one-item measure – the anxiety thermometer may simply not have been sensitive enough to detect a statistically significant difference. Indeed, *a posteriori* power analyses with G-Power (Faul et al., 2009) – based on the current sample and analyses and with effect sizes as reported in Table 5 – indicate that statistical power for the anxiety thermometer at *t*2 was insufficient to detect a small effect (i.e., with power = 0.67), whereas power was sufficient (i.e., >0.85) for all other dependent variables.

#### Mindfulness

Our findings largely contradict hypotheses H2a and H4a in that mindfulness was neither significantly associated with nor significantly predicted anxiety in response to the HRSST. The only significant associations that were observed regarded the WAI-S confidence subscale at baseline (see Tables 3, 5), indicating that mindful individuals tended to show slightly lower baseline levels of confidence. Note however, that baseline levels of confidence were generally positive and showed little between-subject variation (see Table 1). As such, the observed effect is likely to be of low clinical significance. Analyses of subscales (see Table 3) suggest that the observed effect is likely driven by the KIMS subscale “*describing*,” which was negatively correlated with the confidence component of state anxiety at *t*1 as well as at *t*3. Participants with a stronger tendency to (explicitly) describe phenomena in their surroundings reported lower confidence before and after the jump.

The general absence of significant effects regarding mindfulness may be explained by the correlational design of the current study and, potentially, insufficient variability in trait mindfulness (as measured with the KIMS), as well as the fact that the current study deliberately examined effects of mindfulness over and above effects of sensation seeking. As can be seen in Table 5, in case of significant effects, substantial variance in outcome measures was often explained by covariates (e.g., trait anxiety) and sensation seeking, leaving little room for mindfulness to make an additional impact. Still, narrative research from extreme sports (Brymer and Schweitzer, 2013; Arijs et al., 2017; Houge Mackenzie and Brymer, 2018) as well as theoretical explanations from other contexts than sports (Mindfulness Stress Buffering Account; Creswell and Lindsay, 2014; Creswell et al., 2019) suggest a link between trait mindfulness and state anxiety. Future studies are advised to consider effects across a broader range of mindfulness, either by contrasting extremes or by implementing tailored mindfulness interventions.

### Physiological Response to the HRSST

#### Sensation Seeking

Our findings contradict hypotheses H1b and H3b, indicating no significant association between SS and salivary cortisol in response to the HRSST. This lack of association is surprising, as a previous study using the same stress induction protocol (Frenkel et al., 2018b) showed that HSS compared to LSS showed significantly smaller cortisol responses. In the broader literature, however, an apparent dissociation between physiological and psychological stress responses is not uncommon (Breivik, 1999a; Kudielka et al., 2009; Campbell and Ehlert, 2012). A review including 49 studies using the Trier Social Stress Test as a psychosocial stressor detected this mismatch in 75% of the studies (Campbell and Ehlert, 2012). This controversy of the results is explained, among others, by inter-individual differences in the degree of psycho-physiological correspondence, or possible mediating factors.

In explaining the observed null-finding, it is important to consider that the current sample consisted exclusively of highly fit and physically active sport science students, whose self-reported fitness level averaged around 70.98 (*SD* = 18.29) on a 0–100 scale. In line with the stressor adaptation hypothesis (CSA hypothesis; Sothmann et al., 1996), which suggests that adaptation to physical stress (e.g., following regular physical exercise) may transfer to include other stressors, several groups of researchers have found reduced psychological and physiological responses to psychological stress in physically active individuals (Klaperski et al., 2013; Zschucke et al., 2015). Matching these observations, the current study showed that – regardless of sensation seeking and mindfulness – increases in anxiety and salivary cortisol following the HRSST were small and, in case of cortisol, non-significant (see “*Time course analyses*” in the Results section). Addressing this issue, future studies on risk sport-specific stress which examine a highly physically active population, are advised to consider additional means to further intensify the stress protocol.

#### Mindfulness

Our findings contradict hypotheses H2b and H4b, indicating no significant associations between mindfulness and physiological stress response measured by salivary cortisol. As with sensation seeking, this null-finding is likely explained by the non-significant increase in cortisol following the HRSST. In addition to increasing the intensity of the stressor, future studies may consider to examine effects in the context of mindfulness- and acceptance-based interventions (e.g., Gardner and Moore, 2004, 2017; Birrer et al., 2012; Frenkel et al., 2018a; Josefsson et al., 2019) as opposed to examining (small) inter-individual differences in trait mindfulness. Although mindfulness training has a long standing tradition in applied sports psychology (Gardner and Moore, 2004, 2017), only few evidence based intervention studies have examined the effects of mindfulness practice on physiological and psychological performance surrogates or on performance outcomes in sports (Bühlmayer et al., 2017; Hoja et al., 2018). One intervention study that did investigate effects of a mindfulness intervention on HPA axis activation reported decreased salivary cortisol levels following mindfulness (John et al., 2011). In this regard, potential implications remain promising.

### Potential, Limitations, and Outlook

To our knowledge, the current study is the first to examine the effects of sensation seeking and trait mindfulness on psychological and physiological responses to a standardized risk sport-specific stressor. A strength of the current study is the application of an experimentally controlled nature and external validity of the stressor, the HRSST, which allows robust examinations of stress responses and realistically mimics stressful situations in high risk sports. Results from the current study, as well as previous work (e.g., Frenkel et al., 2018b) indicate that the HRSST induces a consistent psychological response, which is characterized by robust increases in self-reported state anxiety. On the other hand, physiological responses to the HRSST have been more inconsistent. Salivary cortisol significantly increased after the HRSST in Frenkel et al.’s (2018b) initial validation study, but did not significantly increase in the current study. It is therefore important to further develop the paradigm so that an increase in cortisol can be reliably induced – also in highly fit and physically active populations. Potential considerations include prolonging the task or adding additional (external) stressors such as observation or evaluation.

Regarding the impact of sensation seeking and mindfulness, the current study employed a correlational design. While this informs about natural between-individual variability, stronger effects may be expected by considering extremes or – with regard to mindfulness – employing within-subject manipulations (e.g., mindfulness training; Bühlmayer et al., 2017; Hoja et al., 2018). Still, the current study identified sensation seeking as a significant predictor of individuals’ psychological response to a risk sport-specific stressor (cf. Breivik, 1999a). Moving beyond the immediate context of high risk sports, this finding bears relevance for other high risk contexts and occupations, such as firefighting, policing or the military, where individuals are confronted with similar stressors and threats to their physical integrity (Neria et al., 2000; Nieuwenhuys and Oudejans, 2010, 2011; Meland et al., 2015; Nieuwenhuys et al., 2015; Giessing et al., 2019) and analyses of sensation seeking may potentially contribute to recruitment and selection processes (e.g., Landman et al., 2016).

In the current study, only male participants were included and – hence – potential gender-specific differences in sensation seeking, mindfulness and stress responses, were not taken into account. The decision to include only male participants was deliberate and driven by the fact that females’ cortisol levels can be biased by the menstrual cycle and contraceptives (Kelly et al., 2008) as well as by the fact that climbing also depends on endurance and strength and that these domains differ between the sexes. In extrapolating the current findings to the wider population, these differences should be taken into account.

Building on the current findings, future studies may include a more detailed analysis of individuals’ psychological response to stress and – in context of the appraisal process (Lazarus and Folkman, 1984) – clarify if the observed stress-buffering effects of sensation seeking are caused by the primary or secondary appraisal. In addition, and in light of recent work from clinical psychology (Engel-Yeger et al., 2016; Serafini et al., 2017), analyses of sensory processing patterns could be helpful to further characterize athletes and their vulnerability to stress. Finally, in order to forward understanding of high risk sports performance, it is important to replicate the current findings and contrast observations with those obtained among actual risk sport athletes (e.g., Breivik, 1999a).

## Conclusion

In conclusion, the current study showed that the personality trait sensation seeking may act as a stress “buffer” and significantly reduces individuals’ psychological response (i.e., self-perceived somatic and cognitive state anxiety) to an experimentally controlled, high risk sport-specific stressor. In contrast to our hypotheses, no additional anxiety-reducing effect was observed for trait mindfulness, and neither sensation seeking nor mindfulness could explain observed variance in individuals’ physiological stress response (i.e., salivary cortisol). Because of the far-reaching negative consequences of stress, identifying protective factors to secure and improve the health and performance of people who are exposed to highly demanding and risky situations (e.g., in the context of work or sports) is of critical importance. With regards to the protective influence of sensation seeking and mindfulness, the current study takes a first step in addressing this issue.

## Data Availability

The raw data supporting the conclusions of this manuscript will be made available by the authors, without undue reservation, to any qualified researcher.

## Ethics Statement

The study’s design was approved by the ethical committee of the Faculty of Behavioral and Cultural Studies of Heidelberg University.

## Author Contributions

This manuscript at hand was mutually developed. Each author contributed to the study planning, data analysis, and interpretation with an additional focus on their respective area of competence. All authors contributed crucially in drafting the aim of the study, concretizing the design, and finishing the manuscript, and examined and agreed to the submitted version of the manuscript. JB, R-BH, and MF conducted the experiments. JB was essentially responsible for the statistical analysis with the support of CK and MS. MF interpreted the data, wrote the first draft of the manuscript together with JB, managed the communication between all authors during the development of the manuscript, assumed responsibility for being the corresponding author, and for keeping the co-authors informed of the progress through the editorial review process, the contents of the reviews, and any revisions made.

## Conflict of Interest Statement

The authors declare that the research was conducted in the absence of any commercial or financial relationships that could be construed as a potential conflict of interest.

## Footnotes

1 “Jumping into the rope” is a term belonging to “fall training”. Jumping and falling often is experienced as exciting by beginners. This circumstance was used for stress induction.

2 “As a robustness check, all analyses were also performed with the WAI-T subscales as covariates. With regards to the associations between sensation seeking, mindfulness and our outcome measures, results for these analyses were highly similar to the reported analyses and showed the same significant and non-significant effects. Detailed analysis reports can be obtained from the first author upon request”.
