# Supplementary material for: Mindful Sensation Seeking: An Examination of the Protective Influence of Selected Personality Traits on Risk Sport-Specific Stress
Source: Front Psychol. 2019 Aug 8;10:1719. doi: 10.3389/fpsyg.2019.01719 (PMC6694797; doi:10.3389/fpsyg.2019.01719)
Supplement: Supplementary file 1 [file Table_1.pdf]

Supplement 1

*Correlations of the psychological and physiological predictors with the WAI-T index.*

| Dependent variables | *WAI-T index* |
|---|---|
| *WAI-S som* $t_1$ | .48** |
| *WAI-S cog* $t_1$ | .47** |
| *WAI-S conf* $t_1$ | -.42** |
| *Anxiety thermometer* $t_1$ | .33** |
| *Anxiety thermometer* $t_2$ | .31** |
| *WAI-S som* $t_3$ | .42** |
| *WAI-S cog* $t_3$ | .40** |
| *WAI-S conf* $t_3$ | -.35** |
| $AUC_g$ | .08 |
| $AUC_i$ | .13 |
| Rise | .10 |
| Cortisol $t_4$ | .11 |
| *SS total* | .16 |
| *KIMS index* | .32*** |

Notes. AUCg: Area under the curve with respect to ground; AUCi: Area under the curve with respect to increase; SS = Sensation Seeking; KIMS = Kentucky Inventory of Mindfulness Skills.

* $p < .05$. ** $p < .01$. *** $p < .001$.
